# Hepatic Manifestations in Hematological Disorders

**DOI:** 10.1155/2013/484903

**Published:** 2013-03-31

**Authors:** Jun Murakami, Yukihiro Shimizu

**Affiliations:** ^1^The Third Department of Internal Medicine, Faculty of Medicine, University of Toyama, Toyama 930-0194, Japan; ^2^Gastroenterology Unit, Takaoka City Hospital, Toyama 933-8550, Japan

## Abstract

Liver involvement is often observed in several hematological disorders, resulting in abnormal liver function tests, abnormalities in liver imaging studies, or clinical symptoms presenting with hepatic manifestations. In hemolytic anemia, jaundice and hepatosplenomegaly are often seen mimicking liver diseases. In hematologic malignancies, malignant cells often infiltrate the liver and may demonstrate abnormal liver function test results accompanied by hepatosplenomegaly or formation of multiple nodules in the liver and/or spleen. These cases may further evolve into fulminant hepatic failure.

## 1. Introduction 

Hepatologists or general physicians sometimes encounter hepatic manifestations of various hematologic disorders in daily practice, including various abnormalities in liver function tests or imaging studies of the liver. Some hematologic disorders also mimic liver diseases. While review articles regarding hematologic disorders and liver diseases have been published previously [1–3], we also review more recent topics in this paper.

## 2. Red Blood Cell (RBC) Disorders 

### 2.1. Hemolytic Anemia (HA)

#### 2.1.1. Classification according to the RBC Destruction Site

When the RBC membrane is severely damaged, immediate lysis occurs within the circulation (intravascular hemolysis). In cases of less severe damage, the cells may be destroyed within the monocyte-macrophage system in the spleen, liver, bone marrow, and lymph nodes (extravascular hemolysis) [4–6]. 

#### 2.1.2. Clinical Presentation

Patients with HA typically present with the following findings: rapid onset of anemia, jaundice, history of pigmented (bilirubin) gallstones, and splenomegaly. Mild hepatomegaly can also occur [4].

#### 2.1.3. Liver Function Tests in HA

 In hemolysis, serum lactate dehydrogenase (LDH) levels (specifically the LDH1 and LDH2 isoforms) increase because of lysed erythrocytes [4].Serum aspartate transaminase (AST) levels are also mildly elevated in hemolysis, with the LDH/AST ratio mostly over 30 [7]. Total bilirubin levels can uncommonly exceed 5 mg/dL if hepatic function is normal, except in the case of acute hemolysis caused by sickle cell crisis. Liver dysfunction can also be caused by blood transfusion for anemia in sickle cell disease (SCD) and thalassemia [1, 3]. 

#### 2.1.4. Hemolysis in Liver Disease

Hemolysis can be caused by either abnormalities in the erythrocyte membranes (intrinsic) or environmental (extrinsic) factors. Most intrinsic causes are hereditary, except for paroxysmal nocturnal hemoglobinuria (PNH) or rare conditions of acquired alpha thalassemia [4]. 

Extrinsic HA is caused by immune or nonimmune mechanisms. Extrinsic nonimmune HA is caused by systemic diseases, including some infectious diseases and liver or renal diseases. Various liver diseases may induce HA, and the two major causes of extrinsic HA in patients with liver disease are destruction of RBCs in an enlarged spleen (hypersplenism) and acquired alterations in the red cell membrane (e.g., target cells, acanthocytes, echinocytes, and stomatocytes). Liver diseases, especially those caused by alcohol intoxication, induce severe hypophosphatemia [8–10], which presumably results in low red cell adenosine triphosphate levels, leading to red cell membrane fragility and spheroidicity. These red cells are easily trapped in the spleen because of their reduced deformability. When excess alcohol consumption is the predominant cause, the condition rapidly improves when alcohol consumption is stopped.

Zieve syndrome is a poorly understood entity characterized by fatty liver/cirrhosis, severe upper abdominal and right upper quadrant pain, jaundice, hyperlipidemia, and HA [11–13]. 

### 2.2. Autoimmune HA (AIHA)

AIHA is characterized by increased breakdown of RBCs due to autoantibodies with or without complement activation. Diagnosis of AIHA includes a combination of clinical and laboratory signs of RBC hemolysis together with detection of autoantibodies and/or complement deposition on RBCs detected by the direct antiglobulin test, also known as the direct Coombs test [14]. In more than half of affected patients, AIHA is associated with an underlying disease including some type of infectious disease, immune disorder, or lymphoproliferative disorder (secondary AIHA), whereas other patients do not have any evidence of underlying disorders (idiopathic or primary AIHA) [15].

#### 2.2.1. Liver Function Tests in AIHA

Laboratory findings of AIHA are not different from those of other causes of hemolysis, that is, reduction in serum haptoglobin, indirect bilirubinemia, and elevated levels of serum LDH (I > II predominant) and AST (mostly LDH/AST > 30). Serum total bilirubin uncommonly exceeds 5 mg/dL, and polyclonal hypergammaglobulinemia is often seen.

#### 2.2.2. Liver Failure in AIHA

 Immunoglobulin (Ig)G antibodies (rarely IgM antibodies) generally react with antigens on the RBC surface at body temperature and are thus referred to as “warm agglutinins,” whereas IgM antibodies (rarely IgG type) react with antigens on the RBC surface below body temperature and are thus referred to as “cold agglutinins.” Warm-reacting IgM antibodies may lead to hepatic failure by* in vivo* autoagglutination [16]. A fatal case with primary AIHA presenting as acute liver failure has been reported [16]. The patient experienced recurrent episodes of intravascular hemolysis. Despite corticosteroid therapy, splenectomy, and multiple blood transfusions, the patient eventually succumbed to liver failure. 

### 2.3. PNH

PNH is an uncommon type of acquired hemolysis, which occurs in middle-aged adults [17, 18]. Patients present with dark urine (hemoglobinuria), usually the morning samples. PNH has been proven to be an acquired clonal genetic disease caused by somatic mutation of the X-linked PIG-A gene in hematopoietic stem cells [19].

#### 2.3.1. Clinical Presentation

 The clinical manifestations of PNH are primarily related to abnormalities in the hematopoietic function, HA, a hypercoagulable state, bone marrow hypoplasia or aplasia, and progression to myelodysplastic syndrome or acute leukemia [18].

#### 2.3.2. Diagnosis of PNH

 PNH was indirectly diagnosed formerly on the basis of the sensitivity of PNH red cells to be lysed by complement. The sucrose lysis test is used as a screening test, and diagnosis is confirmed by the Ham acid hemolysis test [20–22]. However, detection of glycosylinositol phospholipid-linked protein deficiency in PNH by flow cytometric analysis has been developed for diagnosis [23]. 

#### 2.3.3. PNH-Associated Liver Disease

 One of the serious complications of PNH is development of a hypercoagulable state and formation of thrombi. Thrombosis in PNH typically occurs in the intracranial, hepatic, or portal vessels. PNH is one of the most common causes of *de novo* presentation of portal vein thrombosis and a rare cause of Budd-Chiari syndrome [24]. 

### 2.4. Sickle Cell Disease (SCD)

SCD is an autosomal recessive genetic disorder resulting from inheritance of the hemoglobin S (Hb S) variant of the *β*-globin chain. The most severe form with homozygosity for Hb S (Hb SS) is called sickle cell anemia (SCA). Less severe forms possess heterozygosity for Hb S and C (Hb SC) or Hb *β*-thalassemia (Hb *β*-thal). The erythrocytes deform to a crescent shape (sickling) prone to hemolysis, often forming clumps in the vasculature (vaso-occlusive crisis), causing organ damages [25]. 

#### 2.4.1. Hepatic Manifestation in SCD

The liver can be affected by the disease with vascular complications from the sickling process. Moreover, multiple transfusions required for treatment could increase the risk of viral hepatitis, iron overload, and development of pigmented gallstones, all of which may contribute to development of a liver disease called “sickle cell hepatopathy” [26–28]. Acute abdominal pain and abnormal liver function tests as well as jaundice can be caused by acute sickle hepatic crisis, sickle cell intrahepatic cholestasis, cholecystitis, and choledocholithiasis with common bile duct obstruction. 

#### 2.4.2. Liver Function Tests in SCD

 Liver function test abnormalities are common in patients with SCD. Elevation in indirect bilirubin, LDH, and AST without other evidence of liver disease is found in 72% of patients with SCA, which is related to the hemolysis and/or ineffective erythropoiesis [29]. Total bilirubin concentrations are usually <6 mg/dL but may double (<15 mg/L) during sickle hepatic crisis [30]. Serum ALT levels may more accurately reflect hepatocyte injury [29]. Serum alkaline phosphatase (ALP), predominantly bone derived, is commonly elevated [31]. 

Acute elevation in serum aminotransferase can be seen with hepatic ischemia in vaso-occlusive crisis, whereas chronic liver dysfunctions are found in 9%–25% of the patients [29, 32], usually caused by coexisting hepatic diseases, such as chronic hepatitis B or C, common bile duct obstruction, or alcohol consumption. 

#### 2.4.3. Hyperammonemia due to Zinc Deficiency in SCD

 Low zinc plasma levels are reported in 44% of SCD patients [33], which may lead to development of encephalopathy due to hyperammonemia in cirrhotic patients with SCA that can be corrected by zinc administration [34].

#### 2.4.4. Liver Imaging Studies in SCD

The CT findings of patients with homozygous SCA reveal diffuse hepatomegaly. The spleen is usually small and atrophic and may have dense calcifications due to repeated splenic infarction. Double heterozygotes (Hb SC and Hb S*β*-thal) usually have splenomegaly and may show infarcts, rupture, hemorrhage, or abscesses of the spleen.

MRI may show decreased signal intensity in the liver and pancreas [35] due to iron deposition in the SCD patients receiving chronic transfusions [36–39]. Abdominal ultrasound can reveal gallstones or increased echogenicity of the liver and pancreas due to iron deposition [37]. 

## 3. Coagulation Disorders

### 3.1. Disseminated Intravascular Coagulation (DIC)

DIC is a systemic process causing both thrombosis and hemorrhage. The pathogenesis of DIC is primarily due to excessive production of thrombin, leading to widespread and systemic intravascular thrombus formation. Major initiating factors are the release or expression of tissue factor secondary to extensive injury to the vascular endothelium or enhanced expression by monocytes in response to endotoxin and various cytokines. The most common causes of DIC are sepsis, trauma and tissue destruction, cancer, and obstetrical complications.

#### 3.1.1. Diagnosis of DIC

 Diagnosis of DIC is suggested by the history and symptoms, thrombocytopenia, and presence of blood smear microangiopathic changes. The diagnosis is confirmed by laboratory tests that demonstrate evidence of both increased thrombus generation (e.g., decreased fibrinogen) as and increased fibrinolysis (e.g., elevated fibrin degradation products or D-dimer).

#### 3.1.2. Hepatic Manifestation in DIC

 Jaundice is common in patients with DIC and may be due to liver injury and increased bilirubin production secondary to hemolysis. In addition, hepatocellular injury may be produced by sepsis and hypotension. Common manifestations of acute DIC, in addition to bleeding, include thromboembolism and dysfunction of the kidney, liver, lungs, and central nervous system. In a series of 118 patients with acute DIC, hepatic dysfunction was found in 19% [38]. Severe liver disease involves decreased synthesis of coagulation factors and inhibitors [39], fibrinolysis, fibrinogenolysis, and elevated levels of fibrin degradation products. Thrombocytopenia may be induced by hypersplenism secondary to portal hypertension. 

### 3.2. The Antiphospholipid Antibody Syndrome (APS)

The antiphospholipid antibody syndrome (APS) or APLA syndrome is characterized by the presence of one of antiphospholipid antibody (aPL) in the plasma and occurrence of any clinical manifestations including venous or arterial thromboses, or pregnancy morbidity. 

#### 3.2.1. Clinical Presentation

 APS occurs either as a primary or secondary from underlying diseases such as systemic lupus erythematosus (SLE). In a series of primary or secondary APS, deep vein thrombosis (DVT) (32%) thrombocytopenia (22%), livedo reticularis (20%), stroke (13%) superficial thrombophlebitis (9%), pulmonary embolism (9%), fetal loss (8%), transient ischemic attack (7%) and hemolytic anemia (7%) are often observed [40], and venous thromboses are more common than arterial thromboses [41, 42]. Although the most common sites where DVT occurs are the calf and the renal veins, hepatic, axillary, subclavian, and retinal veins, cerebral sinuses, and the vena cava may also be involved. 

#### 3.2.2. Hepatic Manifestation in APS

 The liver involvement may include hepatic or portal venous thrombosis, which could result in Budd-Chiari syndrome, hepatic veno-occlusive disease, hepatic infarction, portal hypertension and cirrhosis. [40, 43].

### 3.3. HELLP Syndrome

HELLP syndrome is defined by hemolysis with a microangiopathic blood smear, elevated liver enzymes, and a low platelet count [44]. HELLP syndrome occurs in approximately 1 to 2 per 1000 pregnancies and in 10 to 20 percent of women with severe preeclampsia/eclampsia. 

#### 3.3.1. Clinical Presentation

The most common clinical presentation is abdominal pain [45], nausea, vomiting, and malaise, which may resemble viral hepatitis, particularly if the serums AST and LDH are markedly elevated [46]. Hypertension and proteinuria are present in approximately 85 percent of the cases. Differential diagnosis includes acute fatty liver of pregnancy (AFLP). Prolongation of the prothrombin time activated partial thromboplastin time (aPTT), low glucose and elevated creatinine concentrations are more common in women with AFLP than those with HELLP.

#### 3.3.2. Hepatic Manifestation in HELLP Syndrome

 HELLP syndrome and severe preeclampsia may be associated with hepatic manifestations, including infarction, hemorrhage, and rupture.

## 4. Cryoglobulinemia

### 4.1. Definition and Classification

Precipitates in serum at temperatures below 37°C referred to cryoglobulin (CG). CG consists of immunoglobulin (Ig) and complement components [47], and the cryoglobulinemia refers to the presence of CG in a patient's serum. There are three types of CG according to Brouet classification, which is based on the clonality of Ig [48]. Type I CG (monoclonal Ig) is usually associated with a hematologic malignancy such as Waldenstrom's macroglobulinemia or multiple myeloma. Type II CG (polyclonal and monoclonal Ig) is often secondary to chronic infections such as hepatitic C or human immunodeficiency virus infection. Type III CG (polyclonal Ig) is often secondary to systemic rheumatic diseases.

### 4.2. Clinical Presentation

 Clinical features of Type I CG (monoclonal Ig) include hyperviscosity syndrome due to hematological malignancies. While Type II and III CGs (mixed and polyclonal Ig, resp.) are present with “Meltzer's triad” of palpable purpura, arthralgia, and myalgia, caused by vasculitis in small- to medium-sized vessels [49].

Secondary lymphoproliferative disorders occur in less than 5 to 10 percent of patients in type II CG patients 5 to 10 years after diagnosis [50–52]. The primary malignancies include B cell non-Hodgkin lymphoma, both intermediate-to-high grade lymphoma and low-grade lymphoma such as immunocytoma, mucosa-associated lymphoid tumors, and centrocytic follicular lymphoma. Among patients with hepatitis C-associated type II cryoglobulinemia, the incidence of non-Hodgkin lymphoma is estimated to be 35-fold higher than that in the general population. 

### 4.3. Cryoglobulinemia in HCV Infection

 The pathogenesis of CG has been most studied in chronic HCV infection. B cell hyperactivation may result from HCV infection into B cells via the cell surface protein CD81 [53], chronic, antigen-nonspecific stimulation by macromolecular serum complexes containing HCV, including HCV-IgG and HCV-lipoprotein [54, 55], or from an HCV antigen-specific mechanism [56], resulting in expansion of specific B cell clones expressing the WA idiotype [57] or V(H)1-69 [58]. HCV particles are often found in the CG complexes, but CG development in hepatitis C infection does not necessarily require HCV virion or its components [59]. 

Among patients with HCV infection, the number of circulating regulatory T cells was compared between patients with symptomatic and asymptomatic CG [60], and the mean levels of regulatory T cells were found to be significantly lower in patients with symptomatic HCV-associated CG than asymptomatic subjects.

### 4.4. Hepatic Manifestation of Cryoglobulinemia

 Hepatic manifestations have been reported as hepatomegaly, abnormal liver function tests, or abnormal liver biopsy in up to 90 percent possibly due to chronic hepatitis itself [61].

## 5. Hematological Neoplasms

### 5.1. Classification of Neoplasms of Hematopoietic Origin

Neoplasms derived from hematopoietic and lymphoid tissues are classified according to their morphologic, immunophenotypic, genetic, and clinical features and by the type of originating cell lineage and differentiation stage according to the widely used and accepted World Health Organization classification system of 2001, which was updated in 2008 [62]. 

Myeloid neoplasms include chronic myeloproliferative neoplasms (MPNs), MDS, or acute leukemias with myeloid lineages. Lymphoid neoplasms are divided into acute lymphoblastic leukemia/lymphoma derived from B or T lymphoid progenitors, or ones derived from mature T or B lymphocytes including plasma cells. Histiocytic/dendritic cell neoplasms are derived from antigen presenting cells or tissue macrophages. Rare cases can be unclassifiable to myeloid or lymphoid lineage [62].

## 6. Myeloid Neoplasms 

Chronic MPNs, also called myeloproliferative disorders, classically include chronic myeloid leukemia (CML), polycythemia vera (PV), essential thrombocythemia, and primary idiopathic myelofibrosis. 

### 6.1. CML

CML is an MPN characterized by dysregulated production and uncontrolled proliferation of mature and immature granulocytes with normal morphology. The tumor cells are derived from a pluripotent hematopoietic stem cell having the acquired *BCR-ABL1* fusion gene, usually through translocation between chromosomes 9 and 22, t(9; 22)(q34; q11), referred to as the Philadelphia (Ph) chromosome. *BCR-ABL1* induces leukemogenesis through kinase dependent and independent signaling pathways. The natural history of CML is variable from the chronic phase to the accelerated phase or blast crisis, but the progression process is not fully understood [62].

#### 6.1.1. Clinical Symptoms and Hepatic Manifestation of CML

 At presentation, 20%–50% of patients are asymptomatic. Laboratory findings include leukocytosis with immature cells of the granulocytic series and basophilia, mild anemia, and thrombocytosis. Symptoms include fatigue, malaise, sweating, and weight loss. Abdominal pain and discomfort may occur in the left upper quadrant (sometimes referred to the left shoulder), and early satiety due to splenomegaly with or without perisplenitis and/or splenic infarction may be present. Variable degrees of hepatomegaly are also observed. Tenderness over the lower sternum is sometimes present due to expanding bone marrow, and bleeding episodes due to platelet dysfunction are often encountered [63, 64].

In the chronic phase, approximately 50% of patients with CML show mild to moderate hepatomegaly at presentation, with no liver function abnormalities [65]. At the time of blastic crisis, however, liver sinusoidal infiltration by immature cells may lead to liver enlargement and elevated serum ALP levels [66].

### 6.2. PV

PV is one of the chronic MPNs, and the clinical features include an increased red cell count, splenomegaly, thrombocytosis and/or leukocytosis, thrombotic complications, erythromelalgia, or pruritus. On physical examination, splenomegaly, facial plethora (ruddy cyanosis), and hepatomegaly can be seen in 70%, 67%, and 40% of patients, respectively [67]. Nonpalpable splenomegaly is recognized in most patients on imaging studies [68, 69].

Gastrointestinal complaints are common in PV, with a high incidence of epigastric distress, peptic ulcers, and gastroduodenal erosions on upper endoscopy [70]. These have been attributed to alterations in gastric mucosal blood flow due to altered blood viscosity and/or increased histamine release from tissue basophils, although one study has indicated a high incidence of positivity for infection with *Helicobacter pylori *[70]. While direct liver involvement is uncommon, some patients may present with acute or chronic Budd-Chiari syndrome [71].

### 6.3. Primary Myelofibrosis (PMF)

Primary myelofibrosis (PMF) is a chronic, malignant hematologicdisorder characterized by splenomegaly, leukoerythroblastosis, bone marrow fibrosis, and extramedullary hematopoiesis.

#### 6.3.1. Hepatic Manifestation of PMF

At the time of PMF diagnosis, hepatomegaly is observed in 40%–70% of patients and splenomegaly in at least 90% [72–74]. Hepatosplenomegaly is caused by marked extramedullary hematopoiesis, which may develop after splenectomy, especially in the liver [75, 76]. In a report of 10 patients with PMF, a significant increase in the liver size and serum concentrations of ALP, bilirubin, and/or *γ*-GTP was seen in all of the patients who subsequently developed acute liver failure, resulting in death 3-4 weeks after splenectomy [76]. 

#### 6.3.2. Abnormal Liver Function Tests in PMF

Patients with PMF may have nonspecific laboratory test abnormalities, including elevation in serum concentrations of ALP, LDH, uric acid, leukocyte ALP, and vitamin B12 [77, 78]. Increase in ALP may be due to liver or bone involvement of the disease, while increase in LDH may result from ineffective hematopoiesis. 

### 6.4. MPNs and Portal Vein Thrombosis

MPNs can be an uncommon cause of portal vein tyrosine kinase (V617F) thrombosis with unexplained etiology [79–81]. *JAK2* mutation may be detected in such cases [82, 83].

### 6.5. MPNs and Budd-Chiari Syndrome

A *JAK2* mutation can be found in almost all patients with PV and approximately 50 percent of patients with essential thrombocythemia (ET) or PMF. *JAK2* (V617F) mutations have been described in 26 to 59 percent of patients with Budd-Chiari syndrome without apparent findings of MPNs [84–87]. These findings suggest the presence of occult MPNs in some patients with so-called “idiopathic” Budd-Chiari syndrome. 

## 7. Lymphoid Neoplasms 

### 7.1. Hodgkin Lymphoma (HL)

HL, formerly called Hodgkin's disease, is the first recognized lymphoid tumor, which usually arises in lymph nodes and spreads in a contiguous manner via the lymphatic system. HL is histologically characterized by giant cells called Hodgkin/Reed-Sternberg (H/RS) cells, most of which are transformed Epstein-Barr virus-positive B cells present in a reactive cellular background composed of granulocytes, plasma cells, and lymphocytes.

#### 7.1.1. Hepatic Manifestations of HL

Liver infiltration of malignant cells has been reported in 14% of patients with HL. Hepatomegaly is found in 9% of patients with disease stages I-II and in 45% of patients with stages III-IV [88]. Mild elevation of aminotransferase and moderate elevation of ALP can occur due to tumor infiltration or extrahepatic bile duct obstruction [88]. Cholestasis can be caused by direct infiltration of lymphoma cells, extrahepatic biliary obstruction, viral hepatitis, drug hepatotoxicity, or vanishing bile duct syndrome [89–91]. Approximately 3%–13% of patients with HL present with jaundice [90]. Acute liver failure can be caused by ischemia secondary to compression of the hepatic sinusoids by infiltrating lymphoma cells [92, 93].

### 7.2. Non-Hodgkin Lymphoma (NHL)

NHL has been classified by cell morphology as small to large cell type and according to the natural history of the clinical aggressiveness of the disease as low, intermediate, or high grade.

#### 7.2.1. Hepatic Manifestation of NHL

Lymphoma cell infiltration of the liver with hepatomegaly is more common in NHL than in HL, with 16%–43% of cases showing hepatic involvement [88]. Extrahepatic obstruction is also more common in NHL than in HL, and hepatic infiltration is more common in low-grade B-cell lymphomas than in high-grade lymphomas [94]. Acute hepatic failure can occur in NHL as seen in HL [95], which is caused by sudden ischemia related to massive infiltration of the sinusoids or replacement of liver parenchyma by malignant cells [95]. Although liver involvement in both HL and NHL may present as acute hepatic failure [96–101], liver transplantation should be avoided [102]. 

Acute liver failure due to lymphoma can be suspected in cases of acute onset of hepatic enlargement and lactic acidosis different from other causes of liver failure [2, 103].

#### 7.2.2. Abnormal Liver Function Tests in NHL

Liver function tests of NHL patients show mild to moderate elevation in serum ALP [88]. Elevated level of serum LDH is also often seen in patients with NHL, especially in highly aggressive type such as Burkitt or lymphoblastic lymphoma, reflecting high tumor burden, extensive infiltration of the liver, and coincident immune-mediated HA, which are associated with poor prognosis. 

#### 7.2.3. Imaging Studies of the Liver in NHL

Although diffuse hepatosplenomegaly is commonly observed in patients with indolent lymphomas, liver function is usually preserved in NHL. On the other hand, discrete hepatic masses are more common in the highly aggressive subtypes [104, 105]. However, not all focal liver lesions in patients with NHL are due to lymphoma. In a report of 414 consecutive patients with NHL, only 39% of focal liver lesions detected at disease onset were due to NHL and 58% were benign [106], whereas 74% of lesions detected during followup were due to NHL and 15% were due to a malignancy other than NHL (e.g., hepatocellular carcinoma, metastatic tumor from other secondary malignancy). Ascites may be present and can be chylous in cases of lymphatic obstruction. 

### 7.3. Primary Hepatic NHL

Primary NHL of the liver is a rare condition, accounting for <1% of all extranodal lymphomas. Two-thirds of cases occur in men aged approximately 50 years. Presenting symptoms include abdominal pain, fever, hepatomegaly, and abnormal liver function tests with elevation of LDH higher than that of ALT [107, 108]. The most common histological subtype of primary hepatic NHL is diffuse large B-cell lymphoma, comprising 80%–90% of cases. This disease may present with nodules in the liver or diffuse portal infiltration and sinusoidal spread [109].

Acute liver failure from primary hepatic lymphoma has been treated with liver transplantation and subsequent chemotherapy [110]. Although primary hepatic lymphoma is rare, persistent inflammatory processes associated with HCV infection or autoimmune disease may play a role in the lymphomagenesis of hepatic B cells [111]. 

### 7.4. Primary Hepatosplenic NHL

Primary hepatosplenic diffuse large B-cell lymphoma associated with HCV has been reported [112], and fetal acute liver failure can also occur [113]. Although the etiological role of HCV in lymphoma is unknown, HCV-positive lymphomas tend to arise in extranodal sites, especially in the liver, spleen, or salivary glands where HCV resides and chronic infiltration of lymphocytes occurs. 

### 7.5. Intravascular Diffuse Large B-Cell Lymphoma

Intravascular diffuse large B-cell lymphoma or intravascular lymphoma is an uncommon but important condition in patients with rapidly presenting fever, rash, or ischemic, neurologic, or respiratory signs. With this condition, tumor cells usually evolve exclusively within small vessels in the skin, brain, liver, or lung. Biopsies from these organs are required for a histologic diagnosis. 

Symptoms of fever, night sweats, and weight loss are seen in 55%–85% of B-cell lymphoma patients [114, 115]. The organs affected differ according to the area. In Western countries, symptoms related to the central nervous system (39%) and skin (39%) are mostly commonly experienced [114, 116, 117], whereas those involving the bone marrow (32%), liver (26%), and spleen (26%) are less common. In Asia, symptoms related to involvement of the bone marrow (75%), spleen (67%), and liver (55%) are more common [118–121], whereas those involving the central nervous system (27%) and skin lesions (15%) are less common [122]. Hemophagocytic syndrome has also been reported in a Japanese series (Asian variant) [120]. 

Diagnosis of intravascular large cell lymphoma can be established by random skin biopsy [123] or biopsy of organs suspected to be involved; for example, biopsies of the liver if unexplained abnormal liver function tests are seen, lung if unexplained pulmonary symptoms are present, and brain if unexplained neurological symptoms exist [124–127]. 

### 7.6. Hepatosplenic T-Cell Lymphoma

#### 7.6.1. Clinical Presentation

 Hepatosplenic T-cell lymphoma is a rare type of aggressive NHL associated with patients receiving antitumor necrosis factor-alpha therapy and purine analogues to treat inflammatory bowel disease [128]. 

#### 7.6.2. Hepatic Manifestation of Hepatosplenic T-Cell Lymphoma

Clinical features include hepatosplenomegaly, fever, weight loss, night sweats, pancytopenia, and peripheral lymphocytosis. Liver function tests are elevated in approximately 50% of patients with slight elevation in AST, ALT, or ALP. Serum LDH levels are also elevated in approximately 50% of patients, ranging from mild to extremely high. Immunosuppression, especially of T cells, by antitumor necrosis factor-alpha therapy and purine analogues may increase the risk of this disease [129].

### 7.7. Hemophagocytic Syndrome (HPS)

#### 7.7.1. Clinical Presentation

 HPS is a condition presenting with systemic inflammatory symptoms such as fever, hepatosplenomegaly, cytopenias, and hemophagocytosis in bone marrow, spleen, and lymph nodes [130, 131]. HPS is caused by hypercytokinemia, which is triggered by highly stimulated natural killer and cytotoxic T cells. The underlying disorders include viral infections, usually the Epstein-Barr virus in younger patients, rheumatic disorders, immunodeficiency syndromes, and aggressive lymphomas [132]. An aggressive form of NK-cell lymphoma or intravascular lymphoma of an Asian variant was reported to be complicated by HPS [133]. HPS should be suspected if patients meet at least five of the following eight criteria: fever, splenomegaly, cytopenia, hypertriglyceridemia, low fibrinogen level, hemophagocytosis on bone marrow biopsy, low or absent NK cell activity, or elevated levels of ferritin or soluble IL2 receptor [130]. 

#### 7.7.2. Hepatic Manifestation of HPS

 HPS can cause hepatomegaly, jaundice with cholestasis, moderate transaminase elevation, hyperferritinemia, decreased hepatic synthetic function, and fulminant hepatic failure. Hepatotoxicity is caused by hemophagocytosis in the hepatic sinusoids and portal tracts or by focal hepatocellular necrosis [132]. 

## 8. Leukemia

### 8.1. Acute Leukemia

#### 8.1.1. Clinical Presentation

 Acute leukemias are neoplasms originated from precursors of myeloid or lymphoid lineage (rarely ambiguous lineage). Although ALL is the most common malignancy in children, the incidence is increased also in the elderly. The incidence of AML increases with age and AML is the most common types of adult leukemias.

#### 8.1.2. Hepatic Manifestation of Acute Leukemia

 Although hepatic involvement in acute leukemia is usually mild and silent at the time of diagnosis [134], a postmortem study showed liver infiltration in >95% of acute lymphoblastic leukemia (ALL) cases and up to 75% of acute myeloid leukemia (AML) cases [135]. In ALL, infiltration was confined to the portal tracts, whereas in AML, infiltration was observed in both portal tracts and sinusoids. Massive leukemic cell infiltration of the liver may present as fulminant hepatic failure [136]. In patients with acute leukemia, drug-induced liver injury and bacterial or fungal infections may also affect the liver.

#### 8.1.3. AML and Hepatosplenomegaly

 Palpable organomegaly as a presentation of AML is uncommon, and significant lymph node enlargement is rare in patients with AML. Marked hepatosplenomegaly is also uncommon; however, if present, the patient is likely to have ALL or evolution of AML from a prior myeloproliferative disorder (blast crisis of CML).

### 8.2. ALL in Children

At presentation, several abnormalities, including hepatic dysfunction, coagulation abnormalities, hypercalcemia, hypocalcemia, hyperkalemia, and hyperphosphatemia, may be noted in children with ALL [137]. 

### 8.3. Precursor B-ALL/Lymphoblastic Lymphoma (LBL) in Adults

Precursor B-cell ALL is associated with decrease in normal blood cells caused by replacement of the bone marrow with tumor cells. The clinical presentations of patients include anemia, bleeding tendency, or susceptibility to infections. B-symptoms such as fever, night sweats, and weight loss are often present but may be mild. Hepatomegaly, splenomegaly, or lymphadenopathy can be seen in up to half of the adult patients upon presentation.

### 8.4. Precursor T-ALL/LBL

Precursor T-ALL/LBL originating from thymic precursor T-cells usually occurs in males aged approximately 20 years old. The clinical presentation includes lymphadenopathy (50%) or an anterior bulky mediastinal mass (50%–75%) [138]. Abdominal involvement is rare, but it could be found primarily in the liver and spleen. More than 80% of patients present with stage III or stage IV disease, and almost 50% have B-symptoms and serum LDH levels are usually elevated. Although the bone marrow is frequently normal at presentation, approximately 60% of patients develop bone marrow infiltration and a subsequent leukemic phase indistinguishable from T-cell ALL [139].

### 8.5. Chronic Lymphoid Leukemia (CLL)

#### 8.5.1. Clinical Presentation

Chronic lymphocytic leukemia (CLL) is one of the chronic lymphoproliferative disorders, characterized by a progressive accumulation of monoclonal lymphoid cells. CLL is considered to be identical to small lymphocytic lymphoma (SLL), which is one of the indolent non-Hodgkin lymphomas [62, 140]. CLL is the most common leukemia in Western countries, accounting for approximately 30 percent of all leukemias in the United States. Although CLL lymphocytes resemble normal small lymphocytes in morphology, they are activated clonal B cells at the stage between pre-B and mature B cells. [141–143]. B-CLL lymphocytes are positive for B-cell-associated antigens (CD19, CD20, CD21, and CD23) and CD5 and express extremely low levels of surface membrane immunoglobulins (IgM or both IgM and IgD).

#### 8.5.2. Clinical Staging of CLL

 The natural history of CLL is heterogenous. The staging systems that are widely used to predict patient prognosis and determine the therapeutic strategies are the Rai system [144] and the Binet system [145]. 

#### 8.5.3. Clinical Features of CLL

The most common physical finding is lymphadenopathy, which is present in 50 to 90 percent of the patients. The other lymphoid organ frequently enlarged in CLL is spleen, being palpable in 25 to 55 percent of the cases. 

#### 8.5.4. Hepatic manifestation of CLL

 Patients with CLL often show mild to moderate liver enlargement at the time of initial diagnosis in 15%–25% of cases [145, 146]. The liver is usually only mildly enlarged, ranging from 2 to 6 cm below the right costal margin, with a span of dullness to percussion of approximately 10–16 cm. Upon palpation, the liver is usually nontender and firm with a smooth surface. An enlarged liver in patients with CLL often displays extensive lymphocytic infiltration in the portal tracts with functional impairment of the liver in late stages [147, 148].

### 8.6. Hairy Cell Leukemia (HCL)

#### 8.6.1. Clinical Presentation

Clinical presentation of HCL includes the following [144, 149]: (1) abdominal fullness due to splenomegaly, which may cause spontaneous splenic rupture [150], (2) systemic symptoms such as fatigue, weakness, and weight loss without fever or night sweats, (3) bleeding tendency secondary to severe thrombocytopenia or recurrent infections, and (4) asymptomatic splenomegaly or cytopenias which may be incidentally recognized, and the most common physical sign of HCL is palpable splenomegaly (80%–90% of cases). Massive splenomegaly extending more than 8 cm below the left costal margin is observed in 25% of cases.

#### 8.6.2. Hepatic Manifestation of HCL

Hepatomegaly and lymphadenopathy are not common in HCL, presenting in approximately 20% and 10% of patients, respectively.

#### 8.6.3. Laboratory Findings

Most patients with HCL present with pancytopenia (60%–80%), anemia (85%), and thrombocytopenia and neutropenia (80%). Leukocytosis may be present in 10%–20% of cases. Abnormal liver function tests and hypergammaglobulinemia are seen in 20% of cases. Leukemia cells often infiltrate the liver, in both the portal tracts and sinusoids, and liver enlargement has been observed in up to 40% of patients [151].

## 9. Myeloma and Related Disorder

### 9.1. Multiple Myeloma

#### 9.1.1. Clinical Presentation

Multiple myeloma is one of the neoplasms of plasma cells (i.e., terminally differentiated B cells) and is increasingly frequent with age. It commonly involves bone marrow and produces a monoclonal immunoglobulin and can cause dysfunction or damages of various organs. Most patients with multiple myeloma present with signs or symptoms related to the infiltration of plasma cells into the bone or to kidney damage from excess light chains [152].

#### 9.1.2. Hepatic Manifestation of MM

Hepatomegaly has been observed in 15%–40% of patients and may sometimes be accompanied by splenomegaly [153, 154]. A Mayo clinic series of 1027 cases from this single institution reported relatively rare symptoms and signs of hepatomegaly (4%) and splenomegaly (1%). 

### 9.2. Amyloidosis

#### 9.2.1. Clinical Presentation

 Amyloidosis refers to the extracellular tissue deposition of amyloid fibrils composed of low molecular weight subunits of proteins. Two major common causes of systemic amyloid deposition are AL and AA amyloidosis. Immunoglobulin light chain (AL) amyloidosis (primary amyloidosis) is composed of monoclonal light chains, with or without plasma cell dyscrasias (multiple myeloma and Waldenstrom's macroglobulinemia). AA amyloidosis is composed of fragments of the acute phase reactant called serum amyloid A. AA amyloidosis is typically reactive (secondary) to chronic inflammation. The symptoms in amyloidosis are nonspecific including fatigue and weight loss. Organomegaly and dysfunction of affected organs, including nephrotic syndrome, restrictive cardiomyopathy, peripheral neuropathy, macroglossia, purpura, or a coagulopathy, are often observed [155]. 

#### 9.2.2. Hepatic Manifestation of Amyloidosis

 Hepatomegaly with or without splenomegaly is seen in 70 percent of the patients. A cholestatic pattern with elevated liver enzymes is seen in approximately 25 percent. Hepatic involvement can occur in all types of amyloidosis, and histologically proven liver involvement in systemic amyloidosis is found in 17% to 98% of the patients [156–158]. In hepatic amyloidosis, deposition of AA amyloid is generally seen in vessels, while the non-AA amyloid deposits appear in a mixed pattern in vessels, sinusoidal cells, and portal stroma [159].

Primary hepatic AL amyloidosis is a rare condition. Hepatomegaly and elevated ALP are present in most patients, which could be associated with poor prognosis [160].
